# An exploration of graph metric reproducibility in complex brain networks

**DOI:** 10.3389/fnins.2013.00067

**Published:** 2013-05-13

**Authors:** Qawi K. Telesford, Jonathan H. Burdette, Paul J. Laurienti

**Affiliations:** ^1^Laboratory for Complex Brain Networks, Department of Biomedical Engineering, Wake Forest University School of MedicineWinston-Salem, NC, USA; ^2^Laboratory for Complex Brain Networks, Department of Radiology, Wake Forest University School of MedicineWinston-Salem, NC, USA

**Keywords:** network science, graph theory, brain networks, complex systems, reproducibility, intraclass correlation coefficient

## Abstract

The application of graph theory to brain networks has become increasingly popular in the neuroimaging community. These investigations and analyses have led to a greater understanding of the brain's complex organization. More importantly, it has become a useful tool for studying the brain under various states and conditions. With the ever expanding popularity of network science in the neuroimaging community, there is increasing interest to validate the measurements and calculations derived from brain networks. Underpinning these studies is the desire to use brain networks in longitudinal studies or as clinical biomarkers to understand changes in the brain. A highly reproducible tool for brain imaging could potentially prove useful as a clinical tool. In this review, we examine recent studies in network reproducibility and their implications for analysis of brain networks.

## Introduction

The foundation of **graph theory** arose in the eighteenth century when Leonhard Euler introduced the Königsberg Bridge problem, thus introducing the concept of vertices (or nodes) and edges (or connections) as a way of representing a problem. However, the field known today as **network science**, did not gain widespread popularity until the introduction of small-world networks by Watts and Strogatz, which described a system with regional specialization and efficient global information transfer (Watts and Strogatz, 1998). The development of scale-free networks by Barabási and Albert further expanded the field with their work on hubs, nodes with a high number of connections, and how node connectivity scaled following a power law distribution (Barabási and Albert, 1999; Albert and Barabási, 2002). Both of these concepts, small-world organization and network hubs, have figured prominently in studies of **brain networks**. While early human studies of functional brain networks suggested a scale-free structure (Eguíluz et al., 2005), more recent studies describe brain networks as an exponentially truncated power-law distribution (Gong et al., 2009; Hayasaka and Laurienti, 2010). In addition, studies have found that brain network hubs localize to different areas of the brain (Achard et al., 2006) and are implicated in various disease states, such as Alzheimer's disease (He et al., 2008; Supekar et al., 2008) and schizophrenia (Lynall et al., 2010; Fornito et al., 2012).

As an increasing number of studies are done in brain networks, there is marked interest in validating the measurements derived from brain network data. Graph metric **reproducibility** is considered essential for test-retest purposes. If the metrics derived from networks change significantly from scan to scan, the statistical power of these measurement is greatly decreased, making such analyses unreliable (Deuker et al., 2009; Telesford et al., 2010). The main reason for focusing on reproducibility is the desire to follow graph metrics longitudinally, particularly for detecting abnormalities (Vaessen et al., 2010), drug-treatment effects (Deuker et al., 2009) or potential clinical biomarkers (Wang et al., 2011).

In this review, we explore various studies examining the reproducibility of graph metrics in brain networks for various modalities and conditions. We will discuss the impact of these findings and the implications of using network science for studying the brain. All the studies discussed in this review utilize the ***intraclass correlation coefficient*** (ICC) to assess reproducibility, so we will briefly discuss this statistical method and highlight other statistical tools also used by investigators. We will then look at the reproducibility of specific graph metrics and how particular methodologies (e.g., threshold level, parcellation scheme, etc.) affect reproducibility. Finally, we will summarize the findings and discuss future implications of these findings.

## Statistical analysis

### Graph metric analysis in the brain

**Figure 1:**
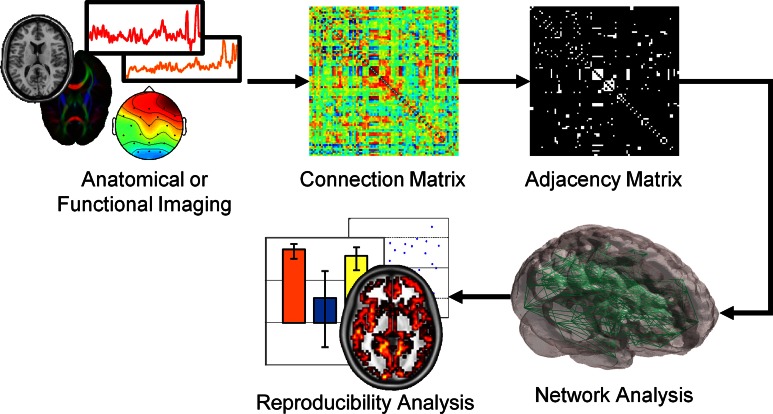
**Schematic of brain network construction and graph metric analysis.** Anatomic or functional data is analyzed to generate a connection matrix, denoting the strength or number of connections between nodes. A threshold is commonly applied to the connection matrix to produce a binary adjacency matrix. From this adjacency matrix, various graph metrics, and statistical analyses can be assessed from these networks.

Brain networks are either derived from anatomic or functional data. In the case of anatomic data, histological samples, diffusion tensor imaging (DTI), or diffusion spectral imaging (DSI) is used to build a network. For DTI/DSI imaging, nodes are defined as voxels in gray matter or gray matter voxels associated with a particular brain region (Hagmann et al., 2008; Vaessen et al., 2010). With each node serving as a seed, probabilistic tractography is used to determine connections between voxels or regions. Similarly, functional networks can be built using functional magnetic resonance imaging (fMRI) (Eguíluz et al., 2005), electroencephalography (EEG) (Micheloyannis et al., 2006; Stam et al., 2007), magnetoencephalography (MEG) (Stam, 2004), and multielectrode array (MEA) data (Srinivas et al., 2007). In functional networks, voxels, sensors or electrodes serve as nodes with links determined by the strong functional coherence of the measured signal. As diagrammed in Figure 1, the anatomic or functional data are used to construct a connection matrix, which can describe the number of connections between two nodes or the correlation between two signals. A threshold is often applied to the correlation matrix and binarized to produce an adjacency matrix. From this matrix, various graph metrics are calculated to determine properties of the network.

### Intraclass correlation coefficient (ICC)

The ICC is a statistic used to measure the absolute agreement between two measurements. It is an appropriate statistic for comparing multiple runs of the same modality because it compares variables that share the same group or category, and measurements that are considered exchangeable (i.e., the order of the measurements does not matter) (McGraw and Wong, 1996; Gonzalez and Griffin, 1999). Reproducibility studies show results in terms of an ICC score where an ICC score of 1 denotes complete agreement, while an ICC score of 0 denotes no agreement. The ICC scores can also be viewed as the level of within-subject variance compared to the between-subject variance; thus, the higher the within-subject variance, the lower the ICC score (Weir, 2005). The interpretation of an ICC score is dependent on several ranges indicating level of agreement: ICC <0.20 indicates poor agreement; 0.21–0.40 indicates fair agreement; 0.41–0.60 indicates moderate agreement; 0.61–0.80 indicates strong agreement; and >0.80 indicates almost perfect agreement (Montgomery et al., 2002). In addition to the ICC score, confidence intervals describe the level of uncertainty of a particular score with wider intervals indicating greater variation between repeated measurements.

There are several variations of the ICC statistic and the appropriate method depends on the form of the data. When testing the reproducibility of *mean* statistics, a one-way model for average measurements, designated ICC(*k*), is used. It is calculated as

$$\text{ICC}(k)=\frac{\left(MS_{B}-MS_{W}\right)}{MS_{B}}$$

where *MS* denotes the mean square (or estimate of variance) from a One-Way ANOVA analysis: *MS*_*B*_ is the mean square between subjects and *MS*_*W*_ is the mean square within subjects (McGraw and Wong, 1996).

To quantify the reproducibility at the nodal level, a one-way model for single measurements, designated ICC(1), is used. It is calculated as

$$\text{ICC}(1)=\frac{MS_{B}-MS_{W}}{MS_{B}+(n-1)MS_{W}}$$

where *n* is the number of subjects, *MS*_*B*_ is the mean square between subjects and *MS*_*W*_ is the mean square within subjects.

### Other reproducibility statistics

While ICC is the popular statistical measure to assess reproducibility, one drawback is that the ICC score is only appropriate for parametric data. To address this issue, distribution-free methods like permutation resampling can be used, providing a method to analyze non-parametric data (Opdyke, 2003; Courrieu et al., 2011). Additional statistics can be used to assess reproducibility of graph metrics; these include Bland-Altman plots and the coefficient of variation (CV). Bland-Altman plots are used to assess repeatability, measuring the difference of means between runs. For repeated measurements, a mean difference of 0 indicates perfect repeatability. Using a one-way analysis of variance with the subjects treated as the factor, the within subject standard deviation (σ_*w*_) is used to create a repeatability coefficient, which denotes the 95% limit of agreement (Bland and Altman, 1999). Similarly, the CV utilizes the within subject standard deviation (σ_*w*_) divided by the overall measurement mean (μ) (Lachin, 2004). The CV indicates the minimum percentage signal change detectable in repeated measures (Vaessen et al., 2010). A summary of the various statistics used to assess reproducibility and corresponding graph metrics can be found in Table 1. For a detailed description of graph metrics and their application to brain networks, see Bullmore and Sporns (2009) and Telesford et al. (2011).

**Table 1:** **Graph metric reproducibility studies**.

**Study**	**Modality**	**Task**	**Reproducibility statistic**	**Graph metrics investigated**
Deuker et al., 2009	MEG	*n*-back (working memory)	ICC	Average mutual information (*MI*)
		Resting state		Clustering coefficient (*C*)
				Path length (*L*)
				Synchronizability (*S*)
				Global efficiency (*E*_glob_)
				Cost efficiency (*CE*)
				Small-world (σ)
				Assortativity (*r*)
				Hierarchy (β)
Vaessen et al., 2010	DTI	N/A	B-A plot	Density
			CV	Nodal strength
			ICC	Nodal diversity
				Edge diversity
				Global connectivity
				Local connectivity
				Global-local
				Dynamics
				Mixing
				Robustness
				Topophysical
				Physical
Telesford et al., 2010	fMRI	Executive function task	B-A plot	Degree (*K*)
			ICC	Clustering coefficient (*C*)
				Path length (*L*)
				Global efficiency (*E*_glob_)
				Local efficiency (*E*_loc_)
Bassett et al., 2011	DSI/DTI	N/A	CV	Clustering coefficient (*C*)
			ICC	Degree (*K*)
			ρ	Path length (*L*)
Schwarz and McGonigle, 2011	fMRI	Resting state	ICC	Clustering coefficient (*C*)
				Assortativity (*r*)
				Local efficiency (*E*_loc_)
				Global efficiency (*E*_glob_)
				Modularity (*Q*)
Wang et al., 2011	fMRI	Resting state	ICC	Connectivity strength
			ρ	Clustering coefficient (*C*)
				Path length (*L*)
				Gamma (γ)
				Lambda (λ)
				Small-world (σ)
				Global efficiency (*E*_glob_)
				Local efficiency(*E*_*loc*_)
				Assortativity (*r*)
				Hierarchy (β)
				Synchronization
				Modularity (*Q*)
				Number of modules
Braun et al., 2012	fMRI	Resting state	ICC	Small-world (σ)
				Clustering coefficient (*C*)
				Local efficiency (*E*_loc_)
				Path length (*L*)
				Global efficiency (*E*_glob_)
				Hierarchy (β)
				Assortativity (*r*)
				Modularity (*Q*)
Liang et al., 2012	fMRI	Resting state	ICC	Clustering coefficient (*C*)
				Path length (*L*)
				Gamma (γ)
				Lambda (λ)
				Small-world (σ)
				Local efficiency (*E*_loc_)
				Global efficiency (*E*_glob_)
				Assortativity (*r*)
				Hierarchy (β)

This table indicates the modality, task, reproducibility statistics, and graph metrics measured to assess reproducibility of graph metrics in brain networks. In every study the intraclass correlation coefficient (ICC) statistic was used; Bland-Altman plots (B-A plot), coefficient of variation (CV) and Pearson's correlation coefficient (ρ) were also used to assess reproducibility.

## Results

### Reproducibility in functional networks

**Figure 2:**
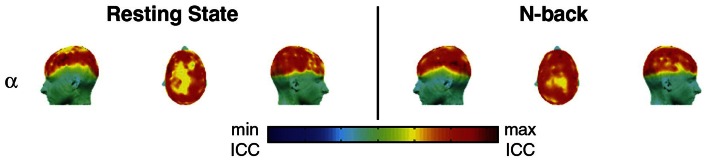
**Reliability (ICC) of network efficiency on a nodal (sensor) level, for the α-band.** While ICC scores were generally low and high during the resting state and *n*-back working memory task, respectively, reproducibility showed spatial variation across the brain. This image was adapted from Deuker et al. (2009).

The first reproducibility study of graph-based brain networks was conducted using MEG data (Deuker et al., 2009). The main goal of this study was to test the reproducibility of graph metrics from MEG recordings. Reproducibility was assessed at the global and nodal level across two MEG recordings during resting state and an *n*-back working memory task. In particular, this study focused on what it called first-order and second-order graph metrics, metrics derived from a single property and multiple properties, respectively (see Table 1 for graph metrics used in studies). Constructing networks from wavelet analysis, global reproducibility was high in lower frequency bands, particularly the α-band during the *n*-back working memory task. However, in the resting state, global reproducibility was poor, except in the α-band, which was high for several metrics. A highlight of this study was that ICC scores were variable across the brain, thus despite the global ICC score, the nodal ICC score could greatly differ (Figure 2). In addition, during task, nodal ICC scores improved as subjects learned the task. The main finding in this study was that reproducibility varied across frequency bands, and showed the highest ICC scores in the lower frequency bands, particularly in the α-band.

**Figure 3:**
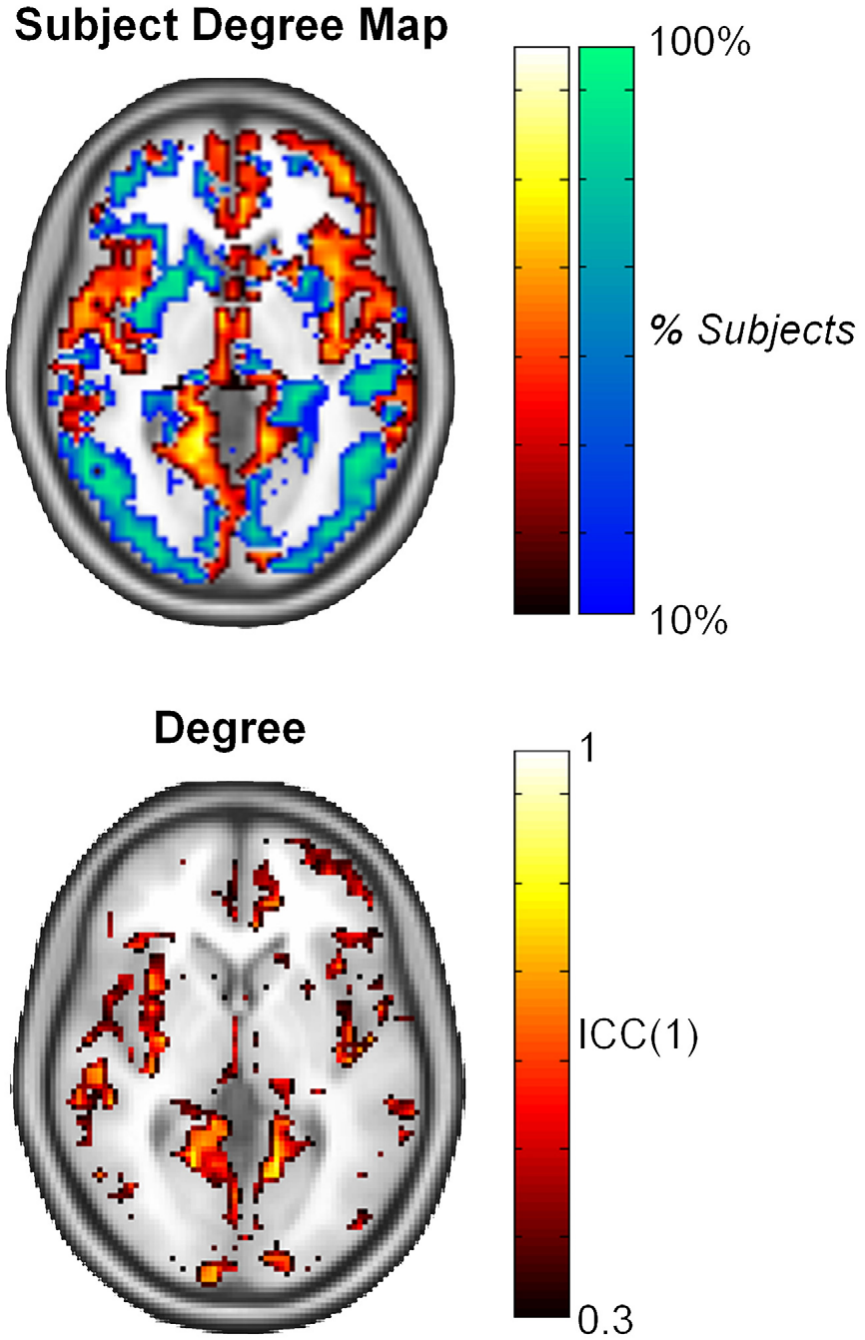
**Subject degree map reflects consistency of high degree nodes (top 25% in orange and yellow) and low degree nodes (bottom 75% in blue and green) across subjects.** ICC scores at the nodal level were found to be consistent with region of high degree in the brain. This image was adapted from Telesford et al. (2010).

Similar findings were reported by Telesford et al. (2010), which investigated reproducibility in voxel-based fMRI networks for an executive function task. High ICC scores for average metrics were found for all graph metrics assessed, except for degree. The distribution of degree follows a truncated power law (Achard et al., 2006; He et al., 2007; Gong et al., 2009; Hayasaka and Laurienti, 2010), and voxel-wise reproducibility showed variation of ICC score across the brain. In particular, it was found that ICC scores were higher in nodes with high degree compared to those with low degree (Figure 3); the link between higher ICC score for nodes with higher degree/strength was also noted in resting state fMRI networks (Wang et al., 2011) and structural networks (Bassett et al., 2011).

Subsequent fMRI network reproducibility studies focused on the resting state (Schwarz and McGonigle, 2011; Wang et al., 2011; Braun et al., 2012; Liang et al., 2012), which found results consistent with the MEG findings by Deuker et al. (2009). In each study, resting state fMRI yielded poor to moderate reproducibility for average metrics; however, depending on the preprocessing steps used the measured ICC score varied considerably. Perhaps the greatest influence on ICC score came from global signal regression. Studies where global signal regression was used reported poor ICC scores (Schwarz and McGonigle, 2011; Wang et al., 2011; Liang et al., 2012), compared to ICC scores when it was not used (Schwarz and McGonigle, 2011; Liang et al., 2012). Additionally, while most studies used the Pearson's correlation coefficient to determine links in the network, partial correlations were also used, but produced lower ICC scores (Liang et al., 2012). In terms of preprocessing, using Pearson's correlation coefficient with regression of signal from white matter and cerebrospinal fluid, six-degree motion parameters, but without global signal regression yielded higher reproducibility (Schwarz and McGonigle, 2011; Liang et al., 2012). Other factors that affected reproducibility was the use of smoothing, which increased ICC scores (Telesford et al., 2010), and sparsity level, which tended to give increased ICC scores for metrics like degree as the network became more dense (Telesford et al., 2010; Braun et al., 2012).

Another topic that received considerable attention in the literature was the inclusion of negative correlations (Schwarz and McGonigle, 2011; Wang et al., 2011; Braun et al., 2012). Schwarz and McGonigle investigated how inclusion of the negative tail affected reproducibility. In addition, different thresholding schemes were investigated. Inclusion of negative correlations was found to decrease reproducibility; this study also reported that utilizing equal thresholds for all subjects yielded higher reproducibility than using the same sparsity for each subject. Nonetheless, as this study noted, using the same threshold for each subject can produce different graphs for each subject, thus the properties across networks can greatly vary (van Wijk et al., 2010). Similar results of low reproducibility when negative correlations were included were also reported by Wang et al. (2011) and Braun et al. (2012). Overall, reproducibility in resting state networks was at best moderate, but generally poor. Despite these results, spatial variation of reproducibility was in line with the reproducibility results reported in task-based fMRI networks.

### Reproducibility in structural networks

While the first study of graph metric reproducibility was conducted using MEG (Deuker et al., 2009), the first structural reproducibility study was done using DTI (Vaessen et al., 2010). This study calculated the ICC score for network metrics between two diffusion scans during the same month using a different number of diffusion gradient directions and a change in gradient amplitude. For average graph metrics, weighted degree and path length showed moderate to strong reproducibility, while clustering coefficient showed more variability for ICC score. However, the number of directions and gradient amplitude did not appear to significantly affect the ICC score for these metrics. The CV values revealed that node degree and clustering coefficient did not exhibit great variability, but connection strength showed more variability for pairs of brain regions. Nonetheless, Bland-Altman plots suggested that these metrics (degree, path length, clustering, and strength) were found to be repeatable for gradient directions and amplitude. The results from the Bland-Altman plots matched those found by Telesford et al. for all metrics except degree, which found degree to be repeatable, but the data was heteroscedastic as the variance increased with the mean (Telesford et al., 2010). However, the differences in these findings may reflect choice of modality (DTI vs. fMRI) or size of the network. The key finding for this study was that while different gradient acquisition schemes did significantly affect the number of long range tracts and density of brain networks, the reproducibility of graph metrics were not affected.

Similar results were reported in a later study by Bassett et al. (2011). In this study, DTI and DSI were done to compare reproducibility for the respective scanning techniques. While both techniques showed high similarity from scan to scan by a Pearson's correlation of the weighted matrix, DTI appeared to show better reproducibility than DSI. DTI also had lower CV values than DSI for most metrics, suggesting that there was less variability for DTI. Similar to findings highlighted in MEG (Deuker et al., 2009) and fMRI (Telesford et al., 2010; Wang et al., 2011), there was nodal/spatial variation in the ICC scores with increased reproducibility reported for nodes of higher strength or degree. Another key finding in this study was simple graph metrics based on a single property were more reproducible than metrics based on multiple properties, which is line with findings by Deuker et al. (2009).

## Discussion

The general finding across these studies suggests variable findings for network reproducibility; however, task-based functional networks have higher reproducibility (Deuker et al., 2009; Telesford et al., 2010) than resting state networks (Schwarz and McGonigle, 2011; Wang et al., 2011; Braun et al., 2012; Liang et al., 2012). Perhaps the biggest influences on reproducibility in resting state networks are preprocessing steps with choice of atlas (Wang et al., 2011), correlation metric (Liang et al., 2012), inclusion or exclusion of negative connections (Schwarz and McGonigle, 2011; Wang et al., 2011), and whether to regress global signal (Schwarz and McGonigle, 2011; Liang et al., 2012).

The choice of graph metric can influence the expected reproducibility. Simpler graph metrics, which depended on a single property, yielded higher ICC scores, while those with multiple properties yielded lower ICC scores (Deuker et al., 2009; Bassett et al., 2011; Braun et al., 2012). Although Telesford et al. only studied simple graph metrics, nodal ICC scores were further found to be influenced by metrics that were degree-dependent (e.g., degree and clustering coefficient), compared to metrics with properties derived from the overall network (e.g., global efficiency and path length) (Telesford et al., 2010). Wang et al. noted that nodes with higher reproducibility were consistent with the default mode network (Wang et al., 2011); as these nodes tend to have higher degree during the resting state (Hagmann et al., 2008), it is likely these results are in line with the degree-dependent reproducibility findings.

Reproducibility measures for graph metrics can certainly be used for simple graph metrics, and sometimes for more complex graph measures. However, for certain measures, such analyses are not suitable, particularly modularity-type analyses. A modularity analysis is a method designed to find the community structure in a network (Newman, 2006). The value of Q gives a sense of how strong the modular structure is in comparison to a random network. However, running modularity analyses multiple times will give a varying number of communities and values for Q. The results of this analysis are a function of the algorithm as opposed to a property of the network. Modularity reproducibility was reported in several studies comparing Q and number of communities (Schwarz and McGonigle, 2011; Wang et al., 2011; Braun et al., 2012); however, one could easily have a network that varies between scans, yet finds the same number of communities with similar Q values. While these networks may be considerably different, a high ICC score in this case would be misleading. Despite the low ICC scores for resting state fMRI for modularity, a more appropriate measure to assess community structure consistency is scaled inclusivity (Steen et al., 2011). However, the subject of quantifying community structure consistency is still a topic requiring further exploration.

Wang et al. devoted much of their study to the comparison of different parcellation schemes for the brain, comparing the AAL atlas, Harvard-Oxford atlas, and a selective ROI-based atlas (Wang et al., 2011). Although the ROI atlas was shown to have the lowest reproducibility, it is important to understand why this parcellation approach should be avoided from a conceptual standpoint. While some studies have shown brain network organization consistent with known functional brain anatomy (Power et al., 2011), such an approach introduces bias into the measured networks. ROI-based networks can identify interactions between specified nodes; however, this selective schema greatly limits interpretation because it neglects brain regions that may exert a greater influence on the network. Even if these ROI-based atlases yield higher reproducibility or match putative functional networks, full brain coverage, as achieved by the AAL atlas, Harvard-Oxford atlas, or voxel-based networks (van Den Heuvel et al., 2008; Hayasaka and Laurienti, 2010) is essential to reliably interpret brain network organization.

Another topic that warrants attention is the focus on individual edges in a network. In several studies, the Pearson's correlation was used in a variety of ways: to show similarity from run to run (Bassett et al., 2011; Wang et al., 2011); for performing ICC analysis on the correlation matrix itself (Wang et al., 2011); and to determine the consistency of edges across subjects (Schwarz and McGonigle, 2011). While these analyses highlight strong edges that appear across a population, focusing on specific edge is misleading when studying **complex systems.** A network represents an interdependent system where edges between nodes are influenced by other nodes in the system. The presence of an edge in one network may be influenced by a specific connectivity pattern, yet this edge may also be present in another network with a different connectivity pattern. In essence, individual edges do not determine the organization of a particular brain network.

While most nodes had low reproducibility, this may reflect inherent differences in connectivity from subject to subject. Despite these inherent differences, certain network topologies, like the default mode network, consistently arise. Since edge relationships are often analyzed to understand network topology, methods that assess differences in community structure differences may be more applicable. The central idea here is that by focusing on individual edges, system interdependence is ignored. Even if most edges have low reproducibility, particular features in a network may still be consistent across the population variability in network topology can still yield the same patterns in a network.

## Conclusion

Network science has become increasingly popular, and the increasing use of graph theory based approaches to neuroimaging has made reproducibility of these networks more important. Generally, reproducibility was found to be moderate or poor for resting state functional networks while task-based functional networks exhibited high reproducibility. Moreover, structural networks tended toward moderate to strong reproducibility. Perhaps the most interesting finding was the spatial variation of reproducibility at the nodal level. Reproducibility appears to have degree/strength dependence, which is useful due to the focus on hub structure in many network studies.

Nevertheless, the inherent problem in all reproducibility studies of the brain lies in the question of knowing truth. Is poor reproducibility a systemic problem with the tools being used, or does the physiological architecture of the brain itself exhibit high variability from run to run? The measurement in a system can be perfectly reproducible, yet physiological changes in the brain can make network metrics less stable. However, it should be noted that when treating the brain as a complex system, it may not be possible to answer such questions with the current tools available. Given the emphasis on independence in many statistical analyses, it is reasonable that a network, being an interdependent system, may require more sophisticated tools of analysis to detect changes within a group or subjects.

### Conflict of interest statement

The authors declare that the research was conducted in the absence of any commercial or financial relationships that could be construed as a potential conflict of interest.

## Key Concept

Graph theory: Field of mathematics that conceives systems as nodes and edges.
Network science: Interdisciplinary field in study of complex systems.
Brain networks: Conception of brain as graph linking areas by structure or function.
Reproducibility: Ability of study to be reproduced.
Intraclass correlation coefficient: Statistic that describes how strongly measurements resemble each other.
Complex systems: A system marked by nonlinear, emergent properties.

## References

[B1] Achard S. Salvador R. Whitcher B. Suckling J. Bullmore E. (2006). A resilient, low-frequency, small-world human brain functional network with highly connected association cortical hubs. J. Neurosci. 26, 63–72 10.1523/JNEUROSCI.3874-05.2006 16399673 PMC6674299

[B2] Albert R. Barabási A.-L. (2002). Statistical mechanics of complex networks. Rev. Mod. Phys. 74, 47–97

[B3] Barabási A.-L. Albert R. (1999). Emergence of scaling in random networks. Science 286, 509–512 10.1126/science.286.5439.509 10521342

[B4] Bassett D. S. Brown J. A. Deshpande V. Carlson J. M. Grafton S. T. (2011). Conserved and variable architecture of human white matter connectivity. Neuroimage 54, 1262–1279 10.1016/j.neuroimage.2010.09.006 20850551

[B5] Bland J. M. Altman D. G. (1999). Measuring agreement in method comparison studies. Stat. Methods Med. Res. 8, 135–160 10.1177/096228029900800204 10501650

[B6] Braun U. Plichta M. M. Esslinger C. Sauer C. Haddad L. Grimm O. (2012). Test–retest reliability of resting-state connectivity network characteristics using fMRI and graph theoretical measures. Neuroimage 59, 1404–1412 10.1016/j.neuroimage.2011.08.044 21888983

[B7] Bullmore E. Sporns O. (2009). Complex brain networks: graph theoretical analysis of structural and functional systems. Nat. Rev. Neurosci. 10, 186–198 10.1038/nrn2575 19190637

[B8] Courrieu P. Brand-D'Abrescia M. Peereman R. Spieler D. Rey A. (2011). Validated intraclass correlation statistics to test item performance models. Behav. Res. 43, 37–55 10.3758/s13428-010-0020-5 21287127

[B9] Deuker L. Bullmore E. T. Smith M. Christensen S. Nathan P. J. Rockstroh B. (2009). Reproducibility of graph metrics of human brain functional networks. Neuroimage 47, 1460–1468 10.1016/j.neuroimage.2009.05.035 19463959

[B10] Eguíluz V. M. Chialvo D. R. Cecchi G. A. Baliki M. Apkarian A. V. (2005). Scale-free brain functional networks. Phys. Rev. Lett. 94:018102 10.1103/PhysRevLett.94.018102 15698136

[B11] Fornito A. Zalesky A. Pantelis C. Bullmore E. T. (2012). Schizophrenia, neuroimaging and connectomics. Neuroimage 62, 2296–2314 10.1016/j.neuroimage.2011.12.090 22387165

[B12] Gong G. He Y. Concha L. Lebel C. Gross D. W. Evans A. C. (2009). Mapping anatomical connectivity patterns of human cerebral cortex using *in vivo* diffusion tensor imaging tractography. Cereb. Cortex 19, 524–536 10.1093/cercor/bhn102 18567609 PMC2722790

[B13] Gonzalez R. Griffin D. (1999). The correlational analysis of dyad-level data in the distinguishable case. Pers. Relatsh. 6, 449–469

[B14] Hagmann P. Cammoun L. Gigandet X. Meuli R. Honey C. J. Wedeen V. J. (2008). Mapping the structural core of human cerebral cortex. PLoS Biol. 6:e159 10.1371/journal.pbio.0060159 18597554 PMC2443193

[B15] Hayasaka S. Laurienti P. J. (2010). Comparison of characteristics between region-and voxel-based network analyses in resting-state fMRI data. Neuroimage 50, 499–508 10.1016/j.neuroimage.2009.12.051 20026219 PMC2824075

[B16] He Y. Chen Z. Evans A. (2008). Structural insights into aberrant topological patterns of large-scale cortical networks in Alzheimer's disease. J. Neurosci. 28, 4756–4766 10.1523/JNEUROSCI.0141-08.2008 18448652 PMC6670444

[B17] He Y. Chen Z. J. Evans A. C. (2007). Small-world anatomical networks in the human brain revealed by cortical thickness from MRI. Cereb. Cortex 17, 2407–2419 10.1093/cercor/bhl149 17204824

[B18] Lachin J. M. (2004). The role of measurement reliability in clinical trials. Clin. Trials 1, 553–566 10.1191/1740774504cn057oa 16279296

[B19] Liang X. Wang J. Yan C. Shu N. Xu K. Gong G. (2012). Effects of different correlation metrics and preprocessing factors on small-world brain functional networks: a resting-state functional MRI study. PLoS ONE 7:e32766 10.1371/journal.pone.0032766 22412922 PMC3295769

[B20] Lynall M.-E. Bassett D. S. Kerwin R. McKenna P. J. Kitzbichler M. Muller U. (2010). Functional connectivity and brain networks in schizophrenia. J. Neurosci. 30, 9477–9487 10.1523/JNEUROSCI.0333-10.2010 20631176 PMC2914251

[B21] McGraw K. Wong S. P. (1996). Forming inferences about some intraclass correlation coefficients. Psychol. Methods 1, 30–46

[B22] Micheloyannis S. Pachou E. Stam C. J. Vourkas M. Erimaki S. Tsirka V. (2006). Using graph theoretical analysis of multi channel EEG to evaluate the neural efficiency hypothesis. Neurosci. Lett. 402, 273–277 10.1016/j.neulet.2006.04.006 16678344

[B23] Montgomery A. Graham A. Evans P. Fahey T. (2002). Inter-rater agreement in the scoring of abstracts submitted to a primary care research conference. BMC Health Serv. Res. 2:8 10.1186/1472-6963-2-8 11914164 PMC101393

[B24] Newman M. E. J. (2006). Modularity and community structure in networks. Proc. Natl. Acad. Sci. U.S.A. 103, 8577–8582 10.1073/pnas.0601602103 16723398 PMC1482622

[B25] Opdyke J. D. (2003). Fast permutation tests that maximize power under conventional Monte Carlo sampling for pair-wise and multiple comparisons. J. Appl. Stat. Methods 2, 27–49

[B26] Power J. D. Cohen A. L. Nelson S. M. Wig G. S. Barnes K. A. Church J. A. (2011). Functional network organization of the human brain. Neuron 72, 665–678 10.1016/j.neuron.2011.09.006 22099467 PMC3222858

[B27] Schwarz A. J. McGonigle J. (2011). Negative edges and soft thresholding in complex network analysis of resting state functional connectivity data. Neuroimage 55, 1132–1146 10.1016/j.neuroimage.2010.12.047 21194570

[B28] Srinivas K. V. Jain R. Saurav S. Sikdar S. K. (2007). Small-world network topology of hippocampal neuronal network is lost, in an *in vitro* glutamate injury model of epilepsy. Eur. J. Neurosci. 25, 3276–3286 10.1111/j.1460-9568.2007.05559.x 17552996

[B29] Stam C. Jones B. Nolte G. Breakspear M. Scheltens P. (2007). Small-world networks and functional connectivity in Alzheimer's disease. Cereb. Cortex 17, 92–99 10.1093/cercor/bhj127 16452642

[B30] Stam C. J. (2004). Functional connectivity patterns of human magnetoencephalographic recordings: a ‘small-world’ network? Neurosci. Lett. 355, 25–28 10.1016/j.neulet.2003.10.063 14729226

[B31] Steen M. Hayasaka S. Joyce K. Laurienti P. (2011). Assessing the consistency of community structure in complex networks. Phys. Rev. E Stat. Nonlin. Soft Matter Phys. 84:016111 10.1103/PhysRevE.84.016111 21867261 PMC3292265

[B32] Supekar K. Menon V. Rubin D. Musen M. Greicius M. D. (2008). Network analysis of intrinsic functional brain connectivity in Alzheimer's disease. PLoS Comput. Biol. 4:e1000100 10.1371/journal.pcbi.1000100 18584043 PMC2435273

[B33] Telesford Q. K. Morgan A. R. Hayasaka S. Simpson S. L. Barret W. Kraft R. A. (2010). Reproducibility of graph metrics in fMRI networks. Front. Neuroinform. 4:117 10.3389/fninf.2010.00117 21165174 PMC3002432

[B34] Telesford Q. K. Simpson S. L. Burdette J. H. Hayasaka S. Laurienti P. J. (2011). The brain as a complex system: using network science as a tool for understanding the brain. Brain Connect. 1, 295–308 10.1089/brain.2011.0055 22432419 PMC3621511

[B35] Vaessen M. J. Hofman P. A. Tijssen H. N. Aldenkamp A. P. Jansen J. F. Backes W. H. (2010). The effect and reproducibility of different clinical DTI gradient sets on small world brain connectivity measures. Neuroimage 51, 1106–1116 10.1016/j.neuroimage.2010.03.011 20226864

[B36] van Den Heuvel M. P. Stam C. J. Boersma M. Hulshoff Pol H. E. (2008). Small-world and scale-free organization of voxel-based resting-state functional connectivity in the human brain. Neuroimage 43, 528–539 10.1016/j.neuroimage.2008.08.010 18786642

[B37] van Wijk B. C. M. Stam C. J. Daffertshofer A. (2010). Comparing brain networks of different size and connectivity density using graph theory. PLoS ONE 5:e13701 10.1371/journal.pone.0013701 21060892 PMC2965659

[B38] Wang J.-H. Zuo X.-N. Gohel S. Milham M. P. Biswal B. B. He Y. (2011). Graph theoretical analysis of functional brain networks: test–retest evaluation on short- and long-term resting-state functional MRI data. PLoS ONE 6:e21976 10.1371/journal.pone.0021976 21818285 PMC3139595

[B39] Watts D. J. Strogatz S. H. (1998). Collective dynamics of ‘small-world’ networks. Nature 393, 440–442 10.1038/30918 9623998

[B40] Weir J. P. (2005). Quantifying test–retest reliability using the intraclass correlation coefficient and the SEM. J. Strength Cond. Res. 19, 231–240 10.1519/15184.1 15705040

